# The G1/S Specific Cyclin D2 Is a Regulator of HIV-1 Restriction in Non-proliferating Cells

**DOI:** 10.1371/journal.ppat.1005829

**Published:** 2016-08-19

**Authors:** Roger Badia, Maria Pujantell, Eva Riveira-Muñoz, Teresa Puig, Javier Torres-Torronteras, Ramón Martí, Bonaventura Clotet, Rosa M. Ampudia, Marta Vives-Pi, José A. Esté, Ester Ballana

**Affiliations:** 1 AIDS Research Institute-IrsiCaixa, Hospital Germans Trias i Pujol, Universitat Autònoma de Barcelona, Badalona, Spain; 2 Health Research Institute Germans Trias i Pujol (IGTP), Hospital Germans Trias i Pujol, Universitat Autònoma de Barcelona, Badalona, Spain; 3 Research Group on Neuromuscular and Mitochondrial Disorders, Vall d’Hebron Institut de Recerca, Universitat Autònoma de Barcelona, and Biomedical Network Research Centre on Rare Diseases (CIBERER), Instituto de Salud Carlos III, Barcelona, Spain; 4 Biomedical Network Research Centre on Diabetes and Associated Metabolic Diseases (CIBERDEM), Instituto de Salud Carlos III, Barcelona, Spain; Universitätklinikum Heidelberg, GERMANY

## Abstract

Macrophages are a heterogeneous cell population strongly influenced by differentiation stimuli that become susceptible to HIV-1 infection after inactivation of the restriction factor SAMHD1 by cyclin-dependent kinases (CDK). Here, we have used primary human monocyte-derived macrophages differentiated through different stimuli to evaluate macrophage heterogeneity on cell activation and proliferation and susceptibility to HIV-1 infection. Stimulation of monocytes with GM-CSF induces a non-proliferating macrophage population highly restrictive to HIV-1 infection, characterized by the upregulation of the G1/S-specific cyclin D2, known to control early steps of cell cycle progression. Knockdown of cyclin D2, enhances HIV-1 replication in GM-CSF macrophages through inactivation of SAMHD1 restriction factor by phosphorylation. Co-immunoprecipitation experiments show that cyclin D2 forms a complex with CDK4 and p21, a factor known to restrict HIV-1 replication by affecting the function of the downstream cascade that leads to SAMHD1 deactivation. Thus, we demonstrate that cyclin D2 acts as regulator of cell cycle proteins affecting SAMHD1-mediated HIV-1 restriction in non-proliferating macrophages.

## Author Summary

Macrophages are a heterogeneous population of immune cells that provide crucial innate immune defense against pathogens, including HIV-1. The molecular biology of HIV-1 infection in macrophages is influenced by the presence of the host cell restriction factor SAMHD1, which is regulated by phosphorylation by cyclin dependent kinases, the catalytic proteins responsible for cell cycle progression. This study shows that differentiation stimuli strongly influence macrophage cell cycle and proliferation characteristics as well as susceptibility to HIV-1 infection through modulation of SAMHD1 activation. We have identified cyclin D2 as the key step controlling susceptibility to HIV-1 infection by modulation of the signaling pathway leading to SAMHD1 phosphorylation. We show that a complex formed by cyclin D2-CDK4-p21 in GM-CSF macrophages is responsible for the lack of the active CDK, which phosphorylates SAMHD1. This situation is reversed in the absence of cyclin D2, leading to the activation of CDKs and subsequent phosphorylation of its substrates, including SAMHD1. Thus, we propose that the differential expression of the G1/S-specific cyclin D2 controls the HIV-1 restriction pathway in primary macrophages.

## Introduction

Macrophages are a highly heterogeneous cell population that plays a prominent role in innate immune system as key effector cells for the elimination of pathogens, infected cells and cancer cells [1, 2]. Macrophages also play an essential role in maintaining tissue homeostasis by supporting tissue development and repairing damaged tissue architecture [1, 3]. Macrophage differentiation from monocytes occurs in the tissue in concomitance with the acquisition of a functional phenotype that depends on microenvironmental signals, accounting for the wide and apparently opposed variety of macrophage functions [4, 5].

Macrophages, as well as other myeloid lineage cells, become susceptible to HIV-1 infection after degradation or inactivation of the restriction factor SAMHD1, a triphosphohydrolase enzyme that controls the intracellular level of dNTPs [6–9]. Phosphorylation of SAMHD1 by cyclin dependent kinases (CDK) has been strongly associated with inactivation of the virus restriction mechanism, providing an association between virus replication and cell proliferation [10–12]. The activity of CDK is regulated by the binding of cyclins, a family of proteins characterized by a periodic, cell-cycle dependent pattern of expression [13, 14]. Cyclin-CDK complexes govern cell cycle progression and proliferation of mammalian cells and thus, pinpoint the specific time in which an event occurs during the cell cycle [13, 14]. We and others have shown that the complex cyclin D3-CDK6 acting upstream of CDK2 controls SAMHD1 phosphorylation and function in primary lymphocytes and macrophages [11, 15–17]. Cyclin-CDK function is also controlled by cyclin dependent kinase inhibitors (CDKIs) that generally act as negative regulators of the cell cycle by binding to CDKs and inhibiting their kinase activity [18]. Of particular importance is p21/waf1, a G1/S phase CDKI, that may also control HIV-1 replication through SAMHD1 [19, 20].

D-type cyclins (cyclins D1, D2 and D3) are regarded as essential links between cell environment and the core cell cycle machinery. D-type cyclins drive cells through the G1 restriction point and into the S phase, after which growth factor stimulation is no longer essential to complete cell division [21]. D-type cyclins share the capacity to activate both CDK4 and CDK6 [14]. Studies on single, double and triple cyclin D knockout mice revealed that D-type cyclin complexes have redundant functions. However, different D-type cyclins exhibit distinct expression patterns depending on the cell type, indicating that each D-type cyclin has essential functions in particular settings, as suggested by the narrow and tissue-specific phenotypes of the knockout mice (reviewed in [21]).

Here, we have used primary human monocyte-derived macrophages (MDMs) differentiated through different stimuli to evaluate macrophage heterogeneity on cell activation and proliferation, characteristics that influence gene and protein expression patterns and determine susceptibility to HIV-1 infection. The comparative study has led to the identification and characterization of a cell cycle dependent pathway that restricts HIV-1 infection in primary macrophages. These non-proliferating macrophage population is characterized by a high expression of the G1/S-specific cyclin D2. Cyclin D2 acts through the binding to CDK4 and p21 in GM-CSF macrophages, a complex which is responsible for the lack of the active CDK that phosphorylates SAMHD1. Data from mouse peritoneal macrophages confirmed the existence of cyclin D2 expressing macrophages in vivo, further supporting the key role of cyclin D2.

## Results

### Differentiation stimuli determine cell cycle progression and susceptibility to HIV-1 infection due to SAMHD1 activation in primary macrophages

**Fig 1:**
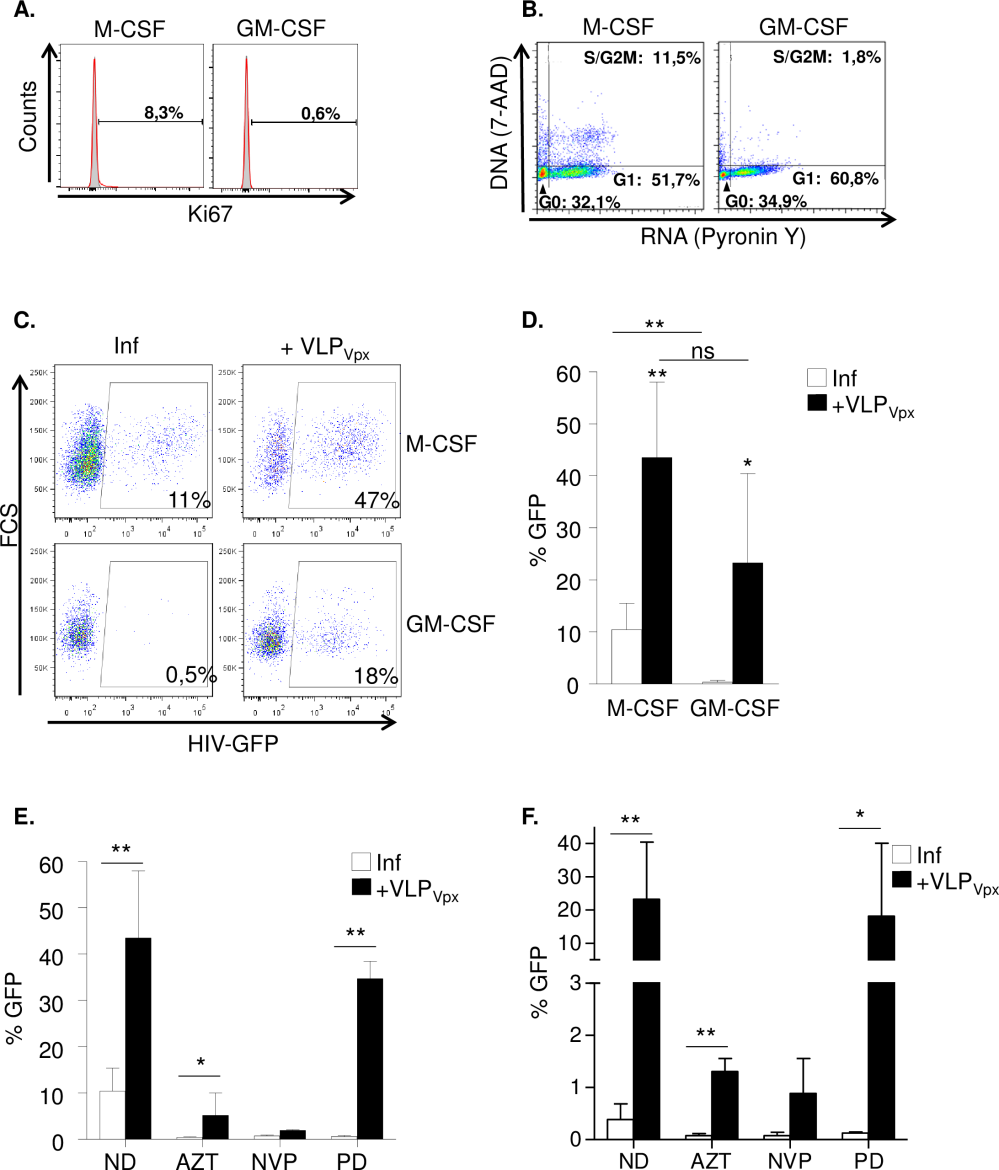
Susceptibility to HIV-1 infection in primary macrophages depends on differentiation stimuli. **(A)** Evaluation of cell proliferation by Ki67 staining. Histograms of a representative donor showing Ki67 staining in M-CSF and GM-CSF derived macrophages. Percentage of positive cells is shown in each case. **(B)** Dot plots representing cell cycle profile showing DNA (7-AAD) and RNA (Pyronin Y) content of M-CSF and GM-CSF differentiated macrophages quantified by flow cytometry. Data from a representative donor is shown. **(C)** Dot plots of infected M-CSF macrophages (upper panels) or GM-CSF macrophages (lower panels) untreated (left) or after treatment (right) with VLP_Vpx._ Data from a representative donor is shown. **(D)** Percentage replication in M-CSF MDM and GM-CSF MDM untreated (white bars) or transduced with VLP_Vpx_ (black bars). Mean ± SD of 4 different donors performed in triplicate is shown. **(E)** and **(F)** Drug sensitivity in M-CSF and GM-CSF differentiated macrophages. Antiviral activity of zidovudine (AZT, 1 μM), nevirapine (NVP, 5 μM) and palbociclib (PD, 4 μM) was evaluated in M-CSF (E) and GM-CSF (F) MDM, untreated (white bars) or transduced with VLP_Vpx_ (black bars). Antiviral potency was decreased with AZT and completely lost with PD after degradation of SAMHD1 with VLP_Vpx_. Mean ± SD of at least 3 different donors performed in triplicate is shown. ND; no-drug, ns; not significant, * p<0.05; ** p<0.005.

Primary monocyte-derived macrophages were differentiated either with M-CSF or GM-CSF, with the aim to characterize differences in cell activation and proliferation patterns and susceptibility to HIV-1 infection. Differentiated macrophages displayed different morphological characteristics dependent on the differentiation stimuli, but no significant differences in cell surface antigen expression or HIV receptor and co-receptors were observed (S1A Fig). Interestingly, cell proliferation and cell cycle patterns were significantly different between macrophage types, i.e., M-CSF macrophages proliferated at higher rates than GM-CSF measured by intracellular Ki67 staining (Fig 1A, 7% *vs*. 0.5% of Ki67+ cells in M-CSF and GM-CSF macrophages respectively, p = 0.02). Similar results were obtained analyzing cell cycle profile by DNA and RNA content staining (Fig 1B), showing higher percentage of cells in S/G2M stage in M-CSF than GM-CSF macrophages.

Susceptibility to HIV-1 infection was also significantly different, being M-CSF macrophages in average roughly 10-fold more susceptible to HIV-1 infection (Fig 1C, left panels and 1D, p = 0.0072), which correlated with higher dNTP levels in cycling M-CSF macrophages compared to GM-CSF as previously reported (S1B Fig and [22, 23]). These results point towards the established link between cell cycle progression and susceptibility to infection as the determinant of the differences observed between macrophage types, a process where SAMHD1 restriction is central [11]. Indeed, degradation of SAMHD1 by HIV-2 Vpx increased HIV replication in both macrophage types, minimizing the initial differences in infection (Fig 1C, right panels, and 1D, p = 0.12). No differences were found in antiviral activity of drugs either targeting viral reverse transcription (AZT or nevirapine) or the cell cycle inhibitor palbociblib [24] (PD, specifically targeting CDK4/6 and inhibiting SAMHD1 phosphorylation) between M-CSF and GM-CSF differentiated macrophages (Fig 1E and 1F). Moreover, after SAMHD1 degradation by HIV-2 Vpx, decreased antiviral potency of AZT and a complete loss of PD antiviral activity were observed, suggesting that cellular events leading to SAMHD1-mediated viral restriction were similar in both types of macrophages [11, 15]. Similar results were obtained when culturing macrophages in the presence of human serum, indicating that the differentiation stimuli induced by the cytokine determines the macrophage phenotype (S2 Fig).

### GM-CSF macrophages show a significant increase in D-type cyclins expression

**Fig 2:**
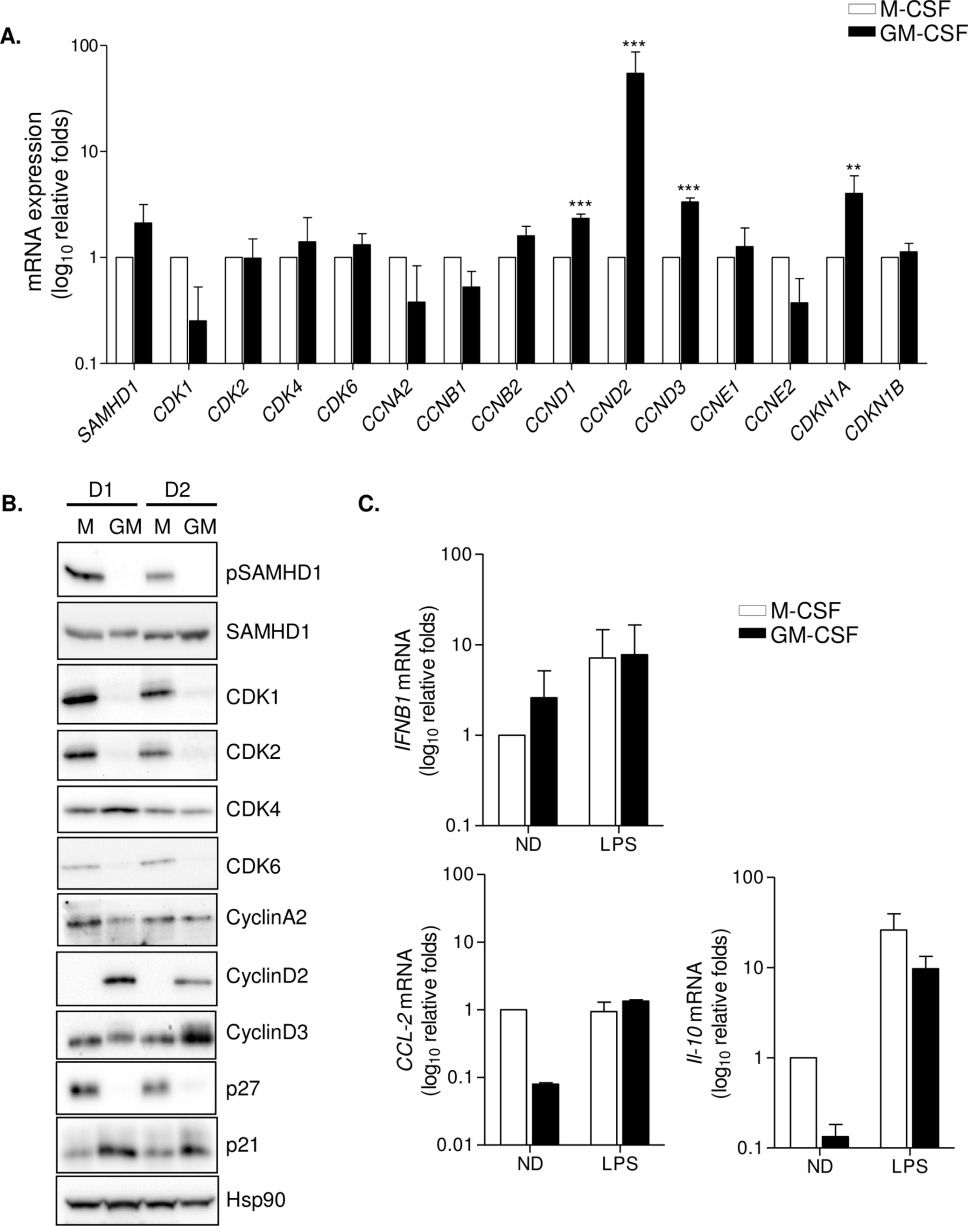
M-CSF and GM-CSF macrophages present differential expression of cell cycle-related proteins and SAMHD1 activation. **(A)** Gene expression of cell cycle-related genes and SAMHD1 restriction pathway. mRNA levels of *SAMHD1*, *CDK1*, *CDK2*, *CDK4* and *CDK6*, the corresponding cyclins (*A2*, *B1*, *B2*, *D1*, *D2*, *D3*, *E1* and *E2*) and CDK2 inhibitor p21 (*CDKN1A*) was quantified in M-CSF (white bars) and GM-CSF (black bars) differentiated macrophages. Data is normalized to M-CSF relative expression. Mean ± SD of 3 independent donors is shown. ** p<0.005; *** p<0.0005. **(B)** Western blot showing protein expression of different cell cycle proteins, SAMHD1 expression and activation and Hsp90 as loading control. Two representative donors are shown. M; M-CSF MDM, GM; GM-CSF MDM. **(C)** Induction of gene expression following LPS (100 ng/ml) treatment of M-CSF and GM-CSF macrophages. Expression of *IFNB1*, *CCL-2* and *IL-10* were evaluated. Data is normalized to untreated M-CSF condition. Mean ± SD of 3 independent donors is shown.

To further investigate the molecular determinants of the observed differences between macrophages types, expression of cell cycle genes implicated in SAMHD1 control were evaluated (Fig 2A). No major gene expression differences were observed, except for a significant upregulation of D-type cyclins (*CCND1*, 2-fold, p = 0.0006; *CCND2*, 40-fold, p = 0.0004; and *CCND3*, 3-fold p = 0.0002) and the CDK inhibitor p21 (*CDKN1A*, 4-fold, p = 0.038) in GM-CSF macrophages (Fig 2A). Analysis of protein expression confirmed the upregulation of cyclin D2, cyclin D3 and p21 in GM-CSF macrophages, and revealed a clear downregulation of CDK protein levels (CDK1, CDK2 and CDK6 but not of CDK4) and the negative cell cycle regulator p27 (Fig 2B). As expected, no differences were found in SAMHD1 expression but a change in SAMHD1 activation was observed, being only phosphorylated and partially inactivated in M-CSF macrophages (Fig 2B, upper panels). Importantly, mouse peritoneal macrophages showed a similar expression pattern than that observed in GM-CSF macrophages, suggesting the existence of cyclin D2 expressing macrophages *in vivo* (S3 Fig). From a functional point of view, stimulation with LPS resulted in the upregulation of *IFNB1*, *IL-10* and *CCL-2* production in both types of macrophages, albeit basal expression levels were different in M-CSF and GM-CSF as reported elsewhere [25, 26] (Fig 2C). These results demonstrate that differentiation stimuli strongly impact the cell cycle profile of primary macrophages, and consequently their capacity to support HIV-1 replication, that may be in part determined by differences in the restriction factor SAMHD1 activation. Differential expression of D-type cyclins, and specially cyclin D2 may represent key regulatory proteins that shape the distinct cell cycle profile and affect HIV-1 restriction.

### D-type cyclins have differential effects on HIV-1 replication depending on the macrophage type

**Fig 3:**
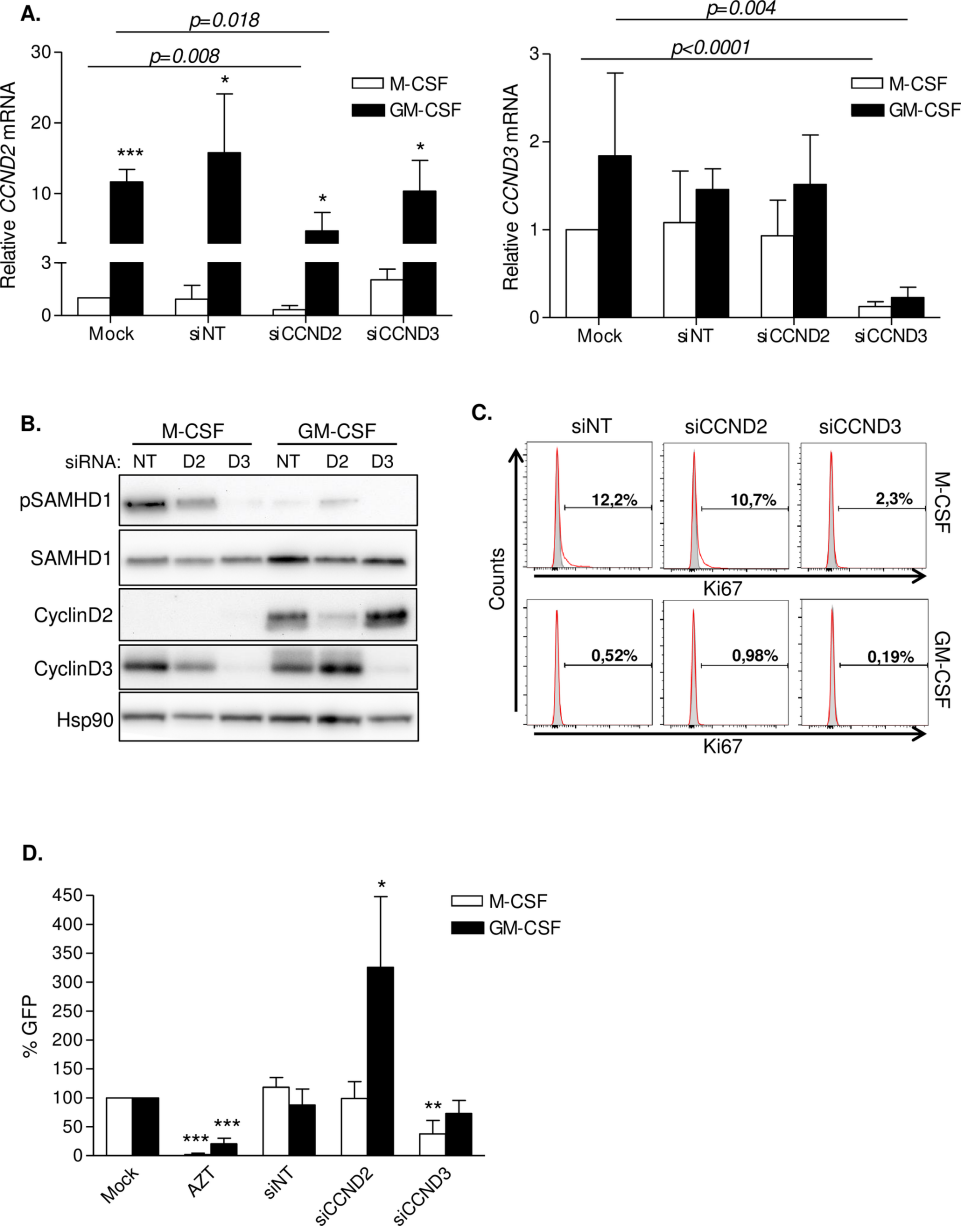
RNAi of cyclin D2 and cyclin D3 differently impact viral replication depending on MDM type. **(A)** Effective and specific knockdown of *CCND2* and *CCND3* expression by siRNA. Relative mRNA expression of *CCND2* (left panel) and *CCND3* (right panel) in M-CSF (white bars) and GM-CSF (black bars) macrophages. mRNA of the corresponding cyclin was measured by quantitative PCR and normalized to GAPDH expression. Data represents mean ± SD of 3 different donors and is normalized to Mock-transfected M-CSF macrophages. **(B)** Protein expression and SAMHD1 activation in cyclin D2 and cyclin D3 knockdown macrophages. Western blot showing cyclin protein expression, SAMHD1 expression and activation in siRNA-treated M-CSF and GM-CSF macrophages. SAMHD1 inactivation is reduced in siCCND3 (D3) M-CSF macrophages and increased in siCCND2 (D2) GM-CSF macrophages compared to the corresponding non-targeting siRNA (NT). Hsp90 was used as loading control. A representative donor is shown. **(C)** Evaluation of cell proliferation in cyclin D2 and cyclin D3 knockdown macrophages. Ki67 staining of siRNA-treated M-CSF (upper panels) and GM-CSF (lower panels) derived macrophages. Histograms from a representative donor are shown. **(D)** HIV-1 replication in siRNA-treated M-CSF or GM-CSF macrophages. Transfected MDM were infected with a VSV-pseudotyped, GFP-expressing HIV-1 and infection measured 72h later by flow cytometry. Data represent percentage replication relative to mock-transfected cells in M-CSF (white bars) or GM-CSF (black bars) macrophages. Mean ± SD of 3 different donors performed in triplicate is shown. * p<0.05; ** p<0.005; *** p<0.0005.

The role of D-type cyclins in macrophage differentiation and HIV-1 susceptibility was further evaluated by RNA interference. Effective and specific downregulation of cyclin D2 and cyclin D3 was achieved at both mRNA and protein level in M-CSF and GM-CSF macrophages (Fig 3A and 3B). Higher expression of *CCND2* in the GM-CSF macrophage population compared to M-CSF, was again clearly observed in all conditions tested (Fig 3A, left panel, and 3B). SAMHD1 expression was not affected by D-type cyclin inhibition, as measured by mRNA level (S4A Fig) or protein expression (Fig 3B). However, different effects on SAMHD1 phosphorylation were observed depending on the targeted cyclin and the differentiation stimuli. As previously reported [16], cyclin D3 knockdown resulted in the abolishment of SAMHD1 phosphorylation in M-CSF macrophages compared to a non-targeting siRNA (Fig 3B, lanes 1–3), an effect that could not be evaluated in GM-CSF macrophages due to low expression levels of phosphorylated SAMHD1. On the contrary, cyclin D2 knockdown resulted in increased SAMHD1 phosphorylation in the GM-CSF macrophage population (Fig 3B, lanes 4–6). Similarly, cyclin D3 but not cyclin D2 knockdown was associated with lower cell proliferation measured as percentage of Ki67 positive macrophages, an effect that was evident in proliferating M-CSF macrophages, but difficult to evaluate in non-proliferating GM-CSF macrophages (Fig 3C).

siRNA-treated macrophages were tested for their capacity to support HIV-1 replication. HIV-1 replication was inhibited after cyclin D3 knockdown in M-CSF macrophages (roughly 60% inhibition, p = 0.0095), whereas inhibition of cyclin D2 expression did not have any effect (Fig 3D, white bars). Conversely, in GM-CSF macrophages cyclin D2 downregulation led to a significant increase of HIV-1 replication (3-fold increase, p = 0.03), but no effect was observed in cyclin D3 knockdown macrophages (Fig 3D, black bars). These results demonstrated different roles for cyclin D2 and D3 at controlling cell proliferation and HIV-1 infection.

### Cyclin D2 expression restricts HIV-1 replication by controlling SAMHD1 activation in GM-CSF macrophages

To determine the molecular bases of cyclin D2 effect on HIV-1 replication in GM-CSF macrophages, most common protein interactions for cyclin D2 were identified by database searches [27, 28]. p21, CDK4 and CDK6 were found as the most common proteins bound to cyclin D2. Cyclin D2 is known to form a complex with CDK4 and CDK6 where it functions as a regulatory subunit of both CDK at G1/S transition [18]. However, CDK6 expression could not be detected in GM-CSF macrophages (Fig 2B), therefore, further characterization was centered in D-type cyclins and their interactors p21 and CDK4.

**Fig 4:**
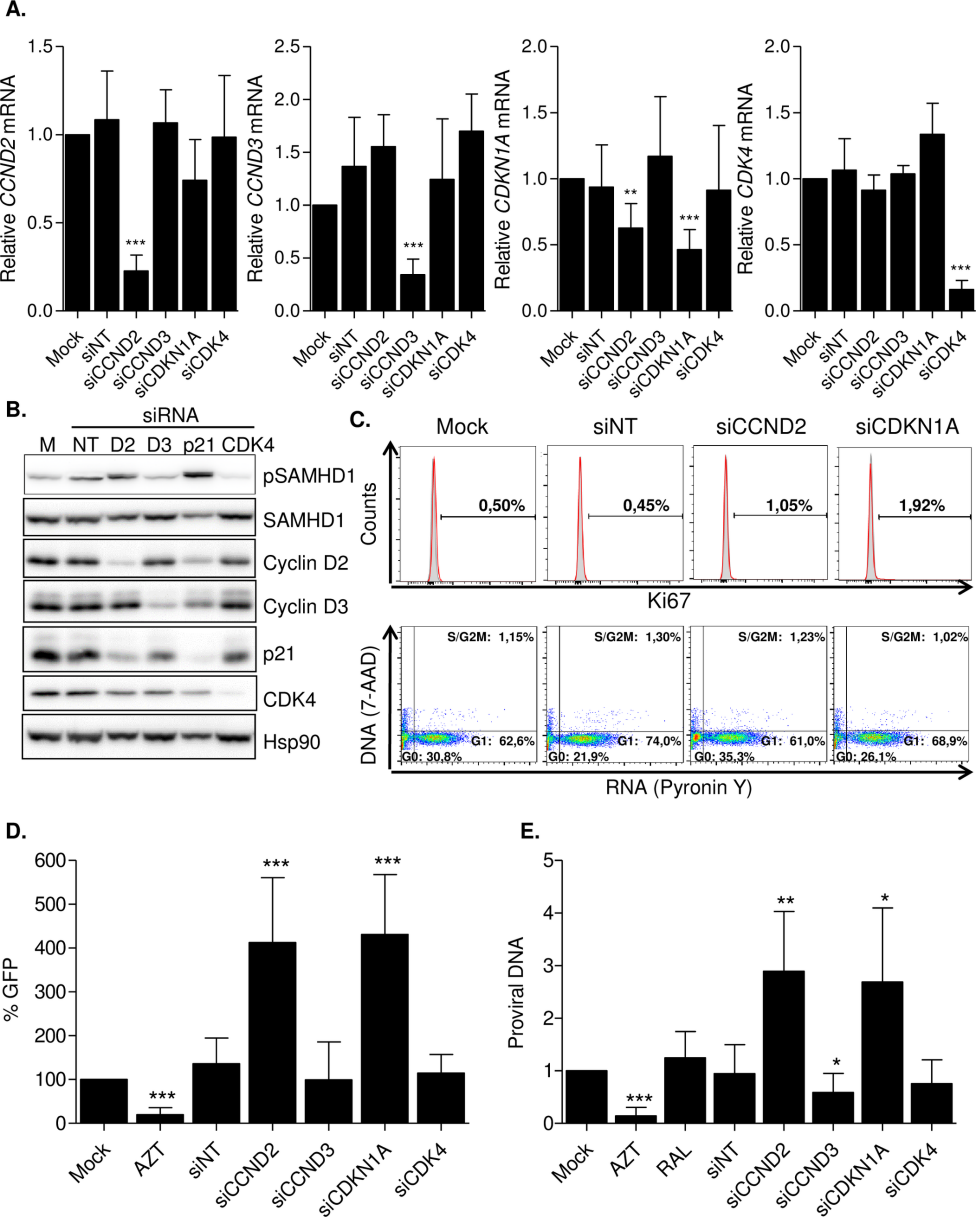
Cyclin D2 expression restricts HIV-1 replication by controlling SAMHD1 activation in GM-CSF macrophages. **(A)** Effective and specific knockdown of *CCND2*, *CCND3*, *CDKN1A* and *CDK4* expression by siRNA in GM-CSF macrophages. mRNA of the corresponding gene was measured by quantitative PCR and normalized to GAPDH expression. Data represents mean ± SD of at least 3 different donors and is normalized to Mock-transfected macrophages. **(B)** Protein expression and SAMHD1 activation in siRNA-treated GM-CSF macrophages. Western blot showing cyclin D2, cyclin D3, CDK4 and p21 protein expression and SAMHD1 expression and activation in siRNA-treated GM-CSF macrophages. SAMHD1 inactivation by phosphorylation is increased in cyclin D2 and p21 knockdown macrophages, compared to Mock-transfected or macrophages treated with a non-targeting siRNA (siNT). **(C)** Evaluation of cell proliferation and cell cycle analysis in siRNA-treated GM-CSF macrophages. Histograms showing Ki67 staining (upper panels) and dot plots representing cell cycle profile by DNA (7-AAD) and RNA (Pyronin Y) staining (lower panels) in siRNA-treated macrophages. Percentage of positive cells in a representative donor is shown in each case. **(D)** HIV-1 replication in siRNA-treated GM-CSF macrophages. Transfected MDM were infected with a VSV-pseudotyped, GFP-expressing HIV-1 and infection measured 72h later by flow cytometry. Data represent percentage replication relative to mock-transfected macrophages. Mean ± SD of at least 3 different donors performed in duplicate is shown. **(E)** Proviral DNA formation after 16h infection with HIV-1 BaL of GM-CSF macrophages transfected with the indicated siRNA or treated with AZT (3 μM) or raltegravir (RAL; 2 μM). Proviral DNA was normalized to mock-treated macrophages. Mean ± SD of at least 3 different donors is shown. * p<0.05; ** p<0.005; *** p<0.0005.

RNA interference was used to effectively and specifically downregulate cyclin D2, cyclin D3, p21 and CDK4 expression in GM-CSF macrophages (Fig 4A and S4B Fig). Evaluation of gene expression in siRNA-treated macrophages suggested that *CCND2* (cyclin D2) and *CDKN1A* (p21) expression is cross-regulated, as interference of *CCND2* expression lead to a significant downregulation of *CDKN1A* (Fig 4A, third panel, 40% inhibition compared to mock macrophages, p = 0.007) and a similar trend was observed when *CCND2* expression was evaluated in siCDKN1A macrophages, although it did not reach statistically significance (Fig 4A, first panel, 30% inhibition compared to mock, p = 0.06). Protein expression confirmed the effective downregulation of all target proteins (Fig 4B) as well as the mutual regulation of cyclin D2 and p21, as knockdown of cyclin D2 or p21 induced a downregulation of p21 or cyclin D2 expression, respectively (Fig 4B, lane 3 and lane 5). As above, SAMHD1 expression was not altered in siRNA-treated macrophages, but significant changes were observed in its phosphorylated form, showing an increased phophorylation in cyclin D2 and p21 knockdown macrophages (Fig 4B, two upper panels). Cell proliferation status showed a small percentage of proliferating cells in all cases, albeit a slight increase in Ki67 positive cells was observed in cyclin D2 and p21 knockdown GM-CSF macrophages (Fig 4C, upper panels). Cell cycle analysis did not show any significant differences between the different macrophages (Fig 4C, lower panels). Altogether, these results reinforce the idea of cyclin D2 and p21 sharing a common regulatory pathway in GM-CSF macrophages.

siRNA-treated GM-CSF macrophages were also tested for their capacity to support HIV-1 replication. Knockdown of cyclin D2 and p21 significantly increased HIV-1 replication in GM-CSF macrophages infected with a VSV-pseudotyped NL4-3 GFP expressing virus (roughly 4-fold increase, p = 0.0007 for siCCND2 and p = 0.0003 for siCDKN1A, respectively, Fig 4D). On the contrary, no effect was seen when cyclin D3 or CDK4 expression were inhibited. Proviral DNA formation was also enhanced in cyclin D2 and p21 knockdown GM-CSF macrophages in short-term infections with the fully replicative HIV-1 R5-tropic strain BaL (roughly 3-fold increase, p = 0.007 for siCCND2 and p = 0.03 for siCDKN1A respectively, Fig 4E). As expected, the HIV-1 reverse transcriptase inhibitor AZT completely blocked proviral DNA formation, while the HIV-1 integrase inhibitor Raltegravir (RAL) did not have an effect on viral DNA formation (Fig 4E). Importantly, confirmatory siRNA sequences targeting Cyclin D2 showed similar effects on infection and proviral DNA formation after HIV-1 BaL infection (S4C and S4D Fig), indicating that cyclin D2 acts as part of a viral restriction mechanism in GM-CSF macrophages. No significant differences in basal cytokine expression and the capacity to induce cytokine expression after LPS stimulation was also preserved in cyclin D2 or p21 knockdown macrophages, suggesting no major functional abnormalities as a result of inhibition of cyclin D2 or p21 (S5 Fig).

### Cyclin D2 associates with p21 and CDK4 in GM-CSF macrophages

**Fig 5:**
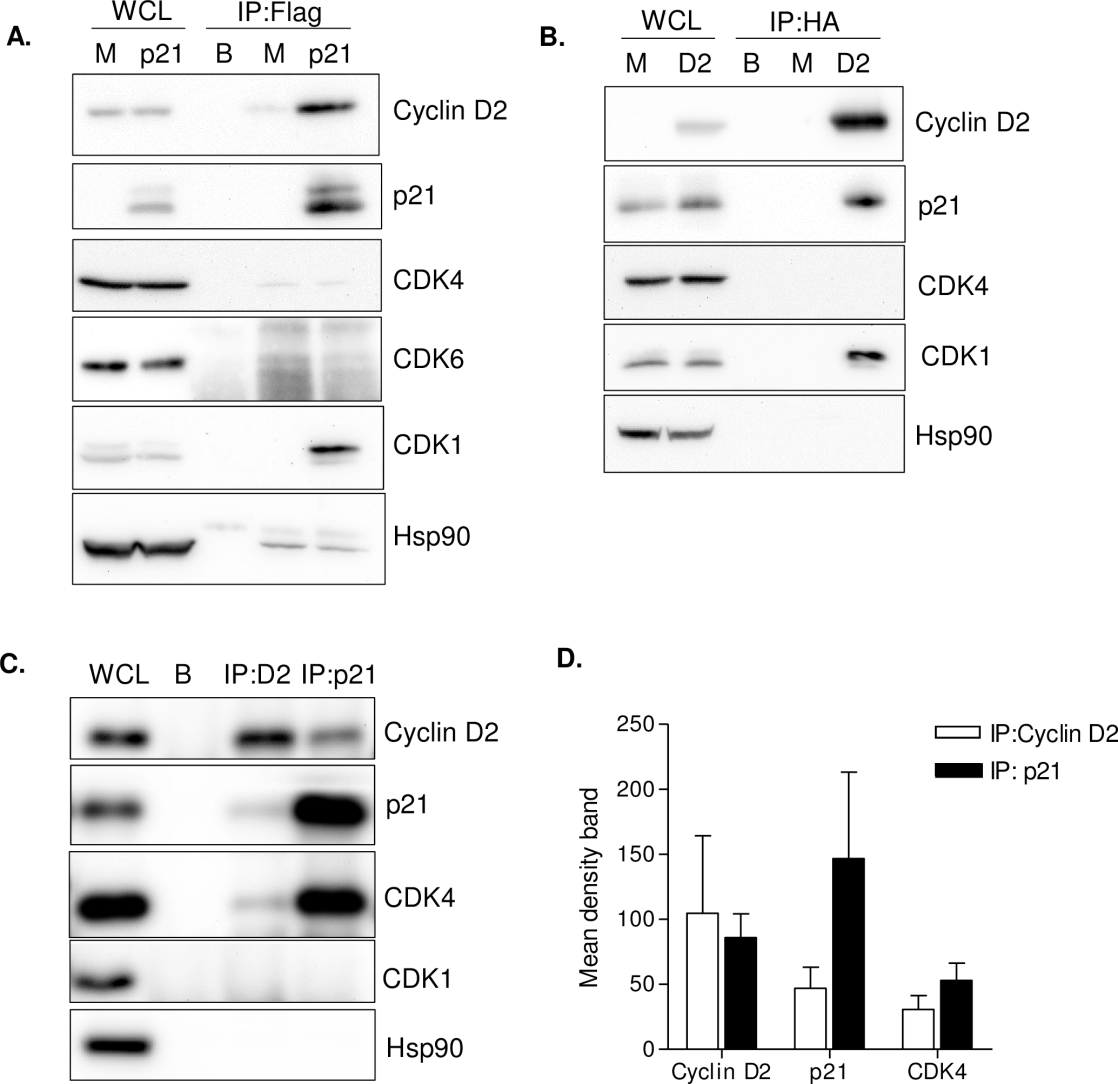
Co-immunoprecipitation (IP) of cyclin D2 with p21. **(A)** Co-IP assay of 293T cells transfected with a plasmid expressing Flag-tagged p21. Lysates from mock- transfected (M) HEK293T cells or transfected with a Flag-p21 expression plasmid (p21) were subjected to immunoprecipitation with anti-Flag antibodies attached to sepharose or sepharose alone (B; beads). Whole cell lysates (WCL) and immunoprecipitates were analyzed by immunoblotting with an anti-cyclin D2 antibody or different CDK antibodies (CDK4, CDK6 and CDK1). Anti-p21 and anti-Hsp90 antibodies were used as controls. **(B)** Co-IP assay of 293T cells transfected with a plasmid expressing HA-tagged cyclin D2. As in (A) Lysates from mock- transfected (M) HEK293T cells or transfected with a HA-cyclin D2 expression plasmid (D2) were subjected to immunoprecipitation with anti-HA antibodies attached to sepharose or sepharose alone (B; beads). Whole cell lysates (WCL) and immunoprecipitates were analyzed by immunoblotting with anti-p21, anti-CDK4 and anti-CDK1 antibodies. Anti-cyclin D2 and anti-Hsp90 antibodies were used as controls. **(C)** Co-IP assay of endogenous cyclin D2 and p21 in GM-CSF primary macrophages. Lysates from primary macrophages where subjected to immunoprecipitation with anti-cyclin D2 or anti-p21 antibodies, attached to sepharose or sepharose attached to IgG alone (B, beads). Whole cell lysate (WCL) and immunoprecipitates were analyzed by immunoblotting with anti-cyclin D2, anti-p21, anti-CDK4 and anti-CDK1 antibodies. Anti-Hsp90 antibody was used as a control. For all Co-IP assays, a representative experiment out of 3 is shown. (**D**) Quantification of endogenous immunoprecipitated proteins with anti-cyclin D2 (white bars) or anti-p21 antibodies (black bars). Mean band density values ± SD of three independent donors is shown.

To investigate the interaction between cyclin D2 and p21, a plasmid expressing a fusion protein Flag-p21 [29] was transfected into HEK293T cells and p21 was immunoprecipitated using Flag-specific agarose beads (Fig 5A). Cyclin D2 co-immunoprecipitated with Flag-p21 (Fig 5A, last lane) and it was not identified when using lysates from mock-transfected cells (M), demonstrating the existence of a protein complex with cyclin D2 and p21. The presence of a CDK in the complex was also investigated and found that CDK1, but not CDK4 or CDK6, immunoprecipitated together with cyclin D2 and p21 in HEK293T cells. The interaction of cyclin D2 and p21 was confirmed by overexpression of a cyclin D2–HA fusion protein [30] in HEK293T cells followed by immunoprecipitation using HA-specific agarose beads. As expected, p21 co-immunoprecipitated with cyclin D2-HA and CDK1 but not CDK4 (Fig 5B), demonstrating the existence of a protein complex between cyclin D2, p21 and a relevant CDK associated to SAMHD1 function.

Deregulation of the cell cycle is a hallmark of most laboratory adapted cell lines in which CDK1 may play a preponderant role in controlling cell proliferation, while other CDKs may have a tissue specific role in driving the cell cycle and cell differentiation [13]. Taking this into account, co-immunoprecipitation experiments of endogenous cyclin D2 and p21 in GM-CSF macrophages were also performed. Importantly, immunoprecipitation of endogenous cyclin D2 or p21 resulted in the identification of p21 or cyclin D2, respectively, demonstrating the coexistence of both proteins in a complex also in GM-CSF primary macrophages (Fig 5C and 5D). The presence of a CDK in the same protein complex was also investigated and CDK4, but not CDK1, was found to co-immunoprecipitate with either cyclin D2 or p21 in primary macrophages (Fig 5C and 5D). When immunoprecipitating p21 higher amounts of cyclin D2 and CDK4 were found than when immunoprecipitating cyclin D2, which might indicate that most p21 is found complexed with cyclin D2 and CDK4, whereas in the case of cyclin D2, only a fraction of the total protein is bound to p21 and CDK4 (Fig 5D). These results further demonstrate the existence of a protein complex formed by cyclin D2, p21 and CDK4 that may govern cell cycle progression and HIV-1 susceptibility in GM-CSF primary macrophages.

### Cyclin D2 expression controls CDK1 and CDK2 activity in GM-CSF macrophages

**Fig 6:**
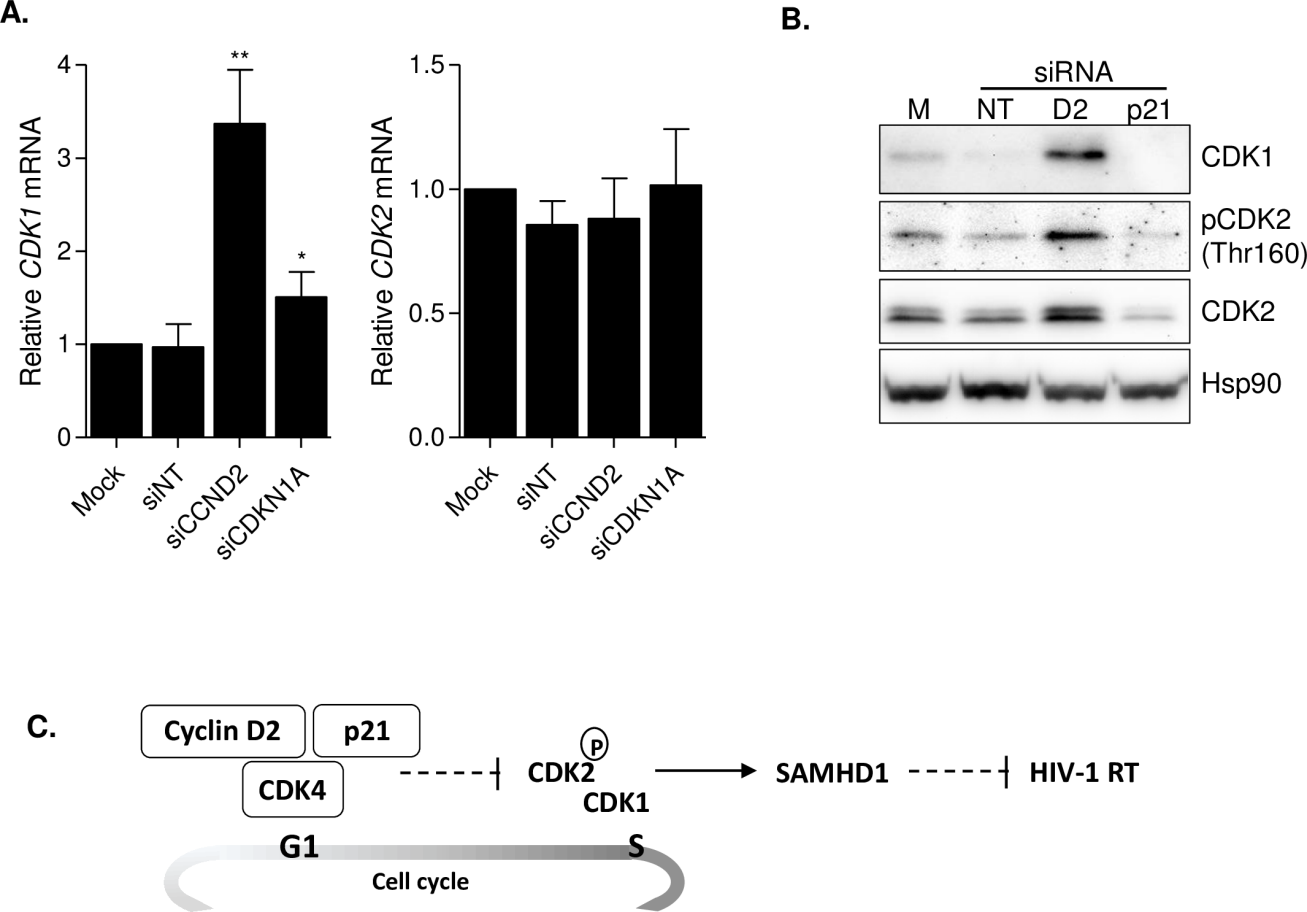
Cyclin D2 expression controls subsequent activation of CDK1 and CDK2. **(A)** CDK1 and CDK2 gene expression in *CCND2* and *CDKN1A* knockdown GM-CSF macrophages. *CDK1* and *CDK2* gene expression was measured by quantitative PCR and normalized to GAPDH expression. Data represents mean ± SD of 3 different donors and is normalized to Mock-transfected macrophages. **(B)** Western blot showing CDK1 and CDK2 expression and phosphorylation of CDK2 in siRNA-treated GM-CSF macrophages. CDK1 expression is upregulated after cyclin D2 knockdown as well as CDK2 activity measured as phosphorylation at Thr130, compared to Mock-transfected or macrophages treated with a non-targeting siRNA (siNT). **(C)** Proposed regulatory model of Cyclin D2 mediated control of HIV-1 restriction in non-proliferating cells. The Cyclin D2/CDK4/p21 complex is responsible for the lack of active CDK2 and CDK1 expression in GM-CSF macrophages. This situation is reversed in the absence of cyclin D2, leading to the activation of CDK2 and CDK1 with the subsequent phosphorylation of its substrates, including SAMHD1.

CDK1 and CDK2 have been identified as the kinases responsible for SAMHD1 phosphorylation in cycling cells and macrophages, respectively [10–12]. Thus, to delineate the molecular pathway regulated by cyclin D2, expression and activation of CDK1 and CDK2 were analyzed in siRNA-treated GM-CSF macrophages (Fig 6). Knockdown of cyclin D2 enhanced significantly CDK1 mRNA (roughly 3-fold, p = 0.0021, Fig 6A, left panel) and protein expression (Fig 6B). Although no significant upregulation of CDK2 expression was observed (Fig 6A, right panel), CDK2 CDK2 regulatory phosphorylation at Thr130 (pCDK2, Fig 6B) was significantly increased suggesting a higher activity of CDK2.

According to the classical model of cell cycle control, CDK4 or CDK6 regulate events in early G0 to G1 phase, CDK2 triggers S phase, CDK2/CDK1 regulate the completion of the S phase and CDK1 is responsible for mitosis [13]. Thus, the complex formed by cyclin D2-CDK4-p21 might be responsible for the lack of active CDK2 and CDK1 expression in GM-CSF macrophages. This situation is reversed in the absence of cyclin D2, leading to the activation of CDK2 and CDK1 with the subsequent phosphorylation of its substrates, including SAMHD1. As a consequence, SAMHD1 phosphorylation results in inactivation of virus restriction and enhancement of HIV-1 replication (Fig 6C).

## Discussion

Macrophages are key components of the innate immune system that reside in tissues, where they function as immune sentinels. Although macrophage heterogeneity has classically been organized around two polarized endpoints known as M1 or classical and M2 or alternative activation [31], recent evidence have revealed an unrecognized greater diversity in the ontogeny and functional diversity of tissue-resident macrophages. It is now established that macrophages from embryonic progenitors can persist in tissues into adulthood and self-maintain by local proliferation (reviewed in [4, 5]). However, monocytes also contribute to the resident macrophage population, on which the local environment can impose tissue-specific macrophage functions (reviewed in [4, 5]). The myeloid colony-stimulating factors, M-CSF and GM-CSF are known to modulate macrophage phenotype and many studies illustrate their importance in the magnitude, duration and character of inflammatory responses [32, 33]. Thus, the description of the distinct molecular phenotypes consequence of M-CSF or GM-CSF stimulation may be important for both acute and chronic inflammatory pathology. Here, we show that differentiation stimuli determine distinct cell proliferation and cell cycle progression in primary macrophages, characteristics that are mostly dependent on the differential expression patterns of cell cycle proteins, especially D-type cyclins, their catalytic partners, CDKs, and the CDK inhibitor p21. Consistent with our observations, upregulation of cyclin D2 in response to GM-CSF treatment has already been reported in different hematopoietic cells [34–36], suggesting that cyclin D2 role might be relevant also for other cell types.

The molecular interplay between the cell cycle, cyclins, and cell function is far from being fully understood. Conceptual advances in the field continue to uncover novel and interesting roles for cyclins in cellular processes that contribute to diseases, such as cancer or pathogenic infections [37]. It is well accepted that cell cycle control plays a major role in determining susceptibility to HIV-1 infection [38, 39]. It has been previously shown that limiting dNTP synthesis, for example through ribonucleotide reductase inhibition by hydroxyurea, limits HIV-1 replication [40]. Thus, differences on cell cycle regulation between M-CSF and GM-CSF macrophages also determined the susceptibility to HIV-1 infection and this may be a direct consequence of the control of the restriction factor SAMHD1 by CDK-mediated phosphorylation. Previous observations in M-CSF macrophages, showed that SAMHD1 phosphorylation was directly phosphorylated by CDK2, whose kinase activity was upstream regulated by the cyclin D3-CDK6 complex [11, 16]. However, the molecular mechanism might be different in GM-CSF macrophages because CDK6 was barely expressed and D-type cyclins expression was significantly upregulated, especially that of cyclin D2. Accordingly, knockdown of cyclin D2 and cyclin D3 resulted in opposite effects depending on the macrophage type, in terms of susceptibility to HIV-1 infection: cyclin D2 restricts infection in non-cycling GM-CSF macrophages and cyclin D3 enables infection in proliferating M-CSF macrophages. All D-type cyclins (D1, D2 and D3) are closely associated to the G1 phase, whose expression is induced by mitogenic signals and therefore play a significant role in cell cycle entry, by assembling together with CDK4 or CDK6 [18, 21, 37]. However, although apparently redundant, single knockout mice exhibit several cell type specific abnormalities [21], suggestive of essential functions on particular settings for each cyclin, explaining, in part, the apparently opposed effects observed here for cyclin D2 and D3 when assessing susceptibility to HIV infection. On the other hand, D-type cyclins are linked to many human malignancies. Cyclin D1 is a well-established oncogene (review in [18]) but cyclin D2 or cyclin D3 overexpression are rarely reported [41, 42]. However, *CCND2* is frequently methylated, with loss of cyclin D2 expression in pancreatic, breast and prostate cancer [43–45], pointing to a potential role as a tumor suppressor rather than an oncogene, indicating that overexpression of cyclin D2 might be limiting cell proliferation, as described here in GM-CSF macrophages.

To uncover the molecular mechanism underlying cyclin D2 function in GM-CSF macrophages and taking into account that cyclins alone do not have catalytic activity *per se*, the study of putative partners of cyclin D2 was addressed. In concordance with the differential gene expression patterns, the inhibition of CDK4 which is similarly expressed in both macrophage types did not change protein profile or HIV-1 susceptibility. Conversely, knockdown of the CDK inhibitor p21, whose expression was also upregulated in GM-CSF macrophages, showed the same effect as that of Cyclin D2, i.e., upregulation of SAMHD1 phosphorylation and enhancement of HIV-1 replication, similar to our previous observation in M-CSF macrophages [20]. p21 belongs to the Cip/Kip family of CDKIs that have historically been considered negative regulators of the cyclin-CDKs and therefore controlling cell cycle progression, especially when referred to cyclin-CDK1 or -CDK2 complexes [20, 46]. However, Cip/Kip family of CDKIs interaction with Cyclin D-CDK4/6 appear much more complex, being involved in both the stabilization of the cyclin D/CDK complex but also acting as inhibitors of its kinase activity [47, 48]. The observation that a complex formed by Cyclin D2/CDK4/p21 was identified in non-cycling GM-CSF macrophages argues in favor of the inhibitory hypothesis at least in this specific cell type. Moreover, the fact that p27, another Cip/Kip family member, is barely expressed in GM-CSF macrophages indicates the specificity to the effect of Cyclin D2/CDK4/p21 complex. A direct inhibition of CDK2 function, similar to that observed in M-CSF [20] cannot be completely rule out, but seems improbable due to the low expression of CDK2 and lack of cell proliferation markers seemed in GM-CSF macrophages.

The present works also highlights the interplay between cell cycle control and viral replication, with important implications that might be broader than simply affecting susceptibility to HIV-1 infection. Interestingly, both p21 and Cyclin D2 were identified as potential markers for viral latency, as both genes showed increased histone modification levels in HIV latently infected cells [49]. These observation suggests that the maintenance or not of HIV-1 latency may also be controlled by cell cycle related proteins such as Cyclin D2 and p21 and thus, open the opportunity for new therapeutic interventions. In addition, p21 and Cyclin D2 were also found upregulated after HTLV-I infection, a process that is mediated by the viral protein Tax, indicating that deregulation of G1/S checkpoint is also relevant for other retroviruses [48].

In summary, the identification of a novel cell cycle-mediated viral restriction pathway in primary non cycling macrophages have provided new evidences of the tight interplay between viral replication and cell cycle control, pointing towards the concerted action of Cyclin D2 and p21 in HIV-1 replication. The demonstration and characterization of specific D-type Cyclin roles in certain cell types, such as that reported for Cyclin D2 here, may also offer a window of opportunity for targeting D Cyclins in viral infections and in human cancers as well, where D-type Cyclin expression is frequently deregulated.

## Materials and Methods

### Cells

PBMC were obtained from buffy coats of blood of healthy donors using a Ficoll-Paque density gradient centrifugation and monocytes were purified using negative selection antibody cocktails (StemCell Technologies) as described before [22]. Monocytes were cultured in complete culture medium (RPMI 1640 medium supplemented with 10% heat-inactivated fetal bovine serum (FBS; Gibco) or human serum (HS; Sigma) and penicillin/streptomycin (Gibco) and differentiated to monocyte derived macrophages (MDM) for 4 days in the presence of monocyte-colony stimulating factor (M-CSF, Peprotech) or granulocyte-macrophage colony-stimulating factor (GM-CSF, Peprotech) both at 100 ng/ml. The protocol was approved by the scientific committee of Fundació IrsiCaixa. Buffy coats were purchased from the Catalan Banc de Sang i Teixits (http://www.bancsang.net/en/index.html). The buffy coats received were totally anonymous and untraceable and the only information given was whether or not they have been tested for disease. When appropriate, differentiated macrophages were incubated with 100 ng/ml of lipopolisaccaride (LPS, Sigma-Aldrich) overnight at 37°C.

TZM cells were received from the National Institutes of Health, AIDS Research and Reference Reagent Program. HEK293T cells were purchased from Dharmacon (Madrid, Spain).

### Mice

Wild-type C57BL/6 inbred mice were purchased from Harlan Laboratories (Sant Feliu de Codines, Barcelona, Spain) and housed at the Animal House facility at the Research Institute Germans Trias i Pujol. Mice were housed under specific pathogen free conditions in a temperature and humidity-controlled room with 12-h light/12-h dark cycle. Only adult males were used in this study. *In vivo* experiments were performed in strict accordance with the recommendations in the Guide for the Care and Use of Laboratory Animals of the Generalitat de Catalunya, Catalan Government and the Principles of laboratory animal care (NIH pub.85–23 revised 1985; http://grants1.nih.gov/grants/olaw/references/phspol.htm). Murine peritoneal macrophages were isolated as described [50]. Briefly, C57Bl/6 mice were sacrificed by cervical dislocation. Immediately after, peritoneal wall was exposed and 10 ml of a cold solution of PBS with 3% FBS per mouse was injected into the peritoneal cavity. Using the same syringe and needle, the fluid from the peritoneum was aspirated, and 8 ml of fluid was recovered. Peritoneal fluid was centrifuged at 4°C and cell pellet was resuspended to adjust cell concentration at 1–3 x 10^6^ cells / ml. To obtain monolayers of peritoneal macrophages, total cells were plated at 2–3 × 10^6^ total nucleated cells/ml in DMEM/F12-10 medium. Cells were allowed to adhere for 1–2 hr at 37°C. Non-adherent cells were removed by gently washing three times with warm PBS. At this time, cells were greater than 90% macrophages and were processed.

### RNA interference

Isolated monocytes were transfected as previously described [11, 16]. Briefly, 50 pmol of the corresponding siRNA (siGENOME SMARTpool from Dharmacon, Thermo-Scientific, Waltham, USA and ThermoFisher Scientific), were transfected using a Monocyte Amaxa Nucleofection kit (Lonza, Basel, Switzerland) following manufacturer instructions. Monocytes were left untreated overnight and then differentiated to macrophages as described above.

### Flow cytometry

For intracellular Ki67 staining, cells were fixed for 3 min with Fixation Buffer (Fix & Perm, Life Technologies) before adding pre-cooled 50% methanol for 10 min at 4°C. Cells were then washed in PBS with 5% FBS and incubated for 30 min with the Ki-67 FITC antibody diluted in permeabilitzation buffer (1:10; clone B56, BD Biosciences). For cell cycle analysis, cell were suspended in 0.03% saponin (Sigma-Aldrich) in PBS and then incubated in 20 mM 7-aminoactinomycin D (7AAD; Sigma-Aldrich) for 30 min at room temperature in the dark, followed by 5 min at 4°C. Then, Pyronin Y (Sigma-Aldrich) was added at a final concentration of 1.5 μg/ml and cells were further incubated at 4°C for 15 min. Flow cytometry was performed in a LSRII flow cytometer (BD Biosciences). The data were analyzed using the FlowJo software (BD Biosciences). To correct the overestimation of G2/M population by miss discrimination of cellular doublets, FL2W versus FL2A of the 7AAD dye was plotted before gating for the distinct cell cycle phases [51].

### Drugs

3-Azido-3-deoxythymidine (zidovudine, AZT) was purchased from Sigma-Aldrich (Madrid, Spain). nevirapine (NVP) and raltegravir (RAL) were obtained from the NIH AIDS Research and Reference Reagent Program. PD-0332991 (palbociclib) was purchased from Selleckchem.

### Quantitative RT-polymerase chain reaction (qRT-PCR)

For relative mRNA quantification, RNA was extracted using the NucleoSpin RNA II kit (Magerey-Nagel), as recommended by the manufacturer, including the DNase I treatment step. Reverse transcriptase was performed using the High Capacity cDNA Reverse Transcription Kit (Life Technologies). mRNA relative levels of all genes were measured by two-step quantitative RT-PCR and normalized to GAPDH mRNA expression using the DDCt method. Primers and DNA probes were purchased from Life Technologies (TaqMan gene expression assays).

Cytokine expression was evaluated by using the commercial TaqMan Human Cytokine Network array (4414255, Life Technologies), which included primers and probes for 28 different cytokine genes. mRNA relative levels of all cytokine genes were measured by two-step quantitative RT-PCR and normalized to GAPDH mRNA expression using the DDCt method.

Intracellular dNTP content was determined using a polymerase-based method [52] as previously described [23].

### Viruses and virus infections

Envelope-deficient HIV-1 NL4-3 clone encoding IRES-GFP (NL4-3-GFP) was pseudotyped with VSV-G by cotransfection of HEK293T cells using polyethylenimine (Polysciences) as previously described [11, 23]. For the production of viral-like particles carrying Vpx (VLP_Vpx_), HEK293T cells were cotransfected with pSIV3+ and a VSV-G expressing plasmid. Three days after transfection, supernatants were harvested, filtered and stored at -80°C. Viral stocks were concentrated using Lenti-X concentrator (Clontech). Viruses were titrated by infection of TZM cells followed by GFP quantification by flow cytometry. R5-tropic HIV-1 strain BaL was grown in stimulated PBMC and specifically titrated for its use in assays of total viral DNA formation in MDM.

M-CSF or GM-CSF differentiated MDM were infected with VSV-pseudotyped NL4-3-GFP and antiviral drugs were added at the time of infection. When necessary, differentiated MDM were pretreated with VLP_Vpx_ for 4h before infection or left with fresh media as a control. Viral replication was measured in all cases two days later by flow cytometry (LSRII, BD Biosciences). Measurement of cell cytotoxicity was performed by flow cytometry, i.e., cells were gated as living or dead, according to flow cytometry FSC and SSC parameters. BaL infections were stopped at 16h to measure only early events of viral infection (reverse transcription). For quantification of proviral DNA, a primer and probe set that is able to amplify both unintegrated and integrated viral DNA was used as described before [11, 16]. DNA was extracted using a DNA extraction kit (Qiagen) and proviral DNA quantifications were performed. Ct values for proviral DNA were normalized using RNaseP as housekeeping gene by the ΔΔCt method. Infections were normalized to an untreated control. To ensure that measured proviral DNA was the product of infection and not result from DNA contamination of the viral stocks samples treated with RT inhibitor AZT (1 μM) were run in parallel. raltegravir (2 μM) was used to ensure that no post-RT steps were being quantified by the assay.

### Western blot

Cells were rinsed in ice-cold phosphate-buffered saline (PBS) and extracts prepared in lysis buffer (50 mM Tris HCl pH 7.5, 1 mM EDTA, 1 mM EGTA, 1 mM Na3VO4, 10 mM Na β-glycerophosphate, 50 mM NaF, 5 mM Na Pyrophosphate, 270 mM sucrose and 1% Triton X-100) supplemented with protease inhibitor (Roche) and 1 mM phenylmethylsulfonyl fluoride. Lysates were subjected to SDS-PAGE and transferred to a PVDF membrane (ImmunolonP, Thermo). The following antibodies were used for immunoblotting: anti-rabbit and anti-mouse horseradish peroxidase-conjugated secondary antibodies (1:5000; Pierce); anti-human Hsp90 (1:1000; 610418, BD Biosciences), anti-SAMHD1 (1:1000; ab67820, Abcam), anti-CDK1 (9116) anti-CDK2 (2546), anti-phosphoCDK2 (Thr160; 2561), anti-CDK4 (D9G3E), anti-CDK6 (3136), anti-cyclin A2 (BF683), anti-cyclin D2 (D52F9), anti-cyclin D3 (DCS22), anti-p21 (2947) and anti-p27 (2552) all 1:1000 from Cell Signaling. Anti-phospho-SAMHD1 Thr592 was obtained by immunization of rabbit using a phosphorylated peptide as described before [53].

### Co-immunoprecipitation assays

HEK293T cells were transfected with Flag-tagged p21 (Addgene plasmid # 16240, gift from Mien-Chie Hung) [29] or HA-tagged Cyclin D2 (Addgene plasmid # 8950, gift from Philip Hinds) [30] expression vectors using lipofectamine 2000 (Invitrogen). 48 h later, cells were chilled to 4°C and cell extracts prepared with lysis buffer as described above. Lysates were cleared by centrifugation at 10500 rpm for 10 min and incubated with anti-FLAG (anti-Flag M2 Affinity Gel, Sigma) or anti-HA (monoclonal anti-HA-agarose, Sigma) antibodies covalently attached to agarose overnight at 4°C on a rocking platform. Beads were then collected by centrifugation at 3000 rpm for 5 min at 4°C, extensively washed in lysis buffer and resuspended in SDS gel loading buffer. The proteins were separated on a 10% SDS-polyacrylamide gel, transferred to a PVDF membrane, and analyzed by immunoblotting with the corresponding antibodies.

Co-immunoprecipitation of endogenously expressed proteins was performed using GM-CSF differentiated macrophages. Cell extracts were prepared as above and lysates were incubated with anti-Cyclin D2 antibody (D52F9, Cell Signaling), anti-p21 antibody (2947, Cell Signalling) or rabbit IgG overnight at 4°C and further incubated with Fast flow Sepharose (Sigma-Aldrich) for 1-2h. Beads were then collected by centrifugation at 3000 rpm for 5 min at 4°C, extensively washed in lysis buffer and resuspended in SDS gel loading buffer. The proteins were separated on a 10% SDS-polyacrylamide gel, transferred to a PVDF membrane, and analyzed by immunoblotting with the corresponding antibodies.

### Statistical methods

Data were analyzed with the PRISM statistical package. If not stated otherwise, all data were normally distributed and expressed as mean ± SD. p-values were calculated using an unpaired, two-tailed, t-student test.

## Supporting Information

S1 Fig Phenotypic characterization of M-CSF and GM-CSF macrophages. **(A)** Summary of cell surface antigens expression. Markers of macrophage differentiation, HIV-1 receptor and coreceptors and cell activation and proliferation markers were evaluated by flow cytometry. The table shows mean ± SD of at least 3 different donors. nd, not detected. **(B)** Intracellular dNTP levels in M-CSF or GM-CSF differentiated MDM. Intracellular dNTPs were extracted from M-CSF (left) or GM-CSF (right) differentiated MDM and dNTP content was determined using a polymerase-based method. dNTP content is highly variable between donors and therefore data is relativized to M-CSF differentiated MDM. Mean ± SD of 3 different donors is shown. * p<0.05
(TIFF)

S2 Fig GM-CSF macrophages cultured with human serum (HS) or fetal bovine serum (FBS) show lower proliferation rates, higher expression of cyclin D2 and are less susceptible to HIV-1 infection compared to M-CSF. **(A)** Evaluation of cell proliferation by Ki67 staining. Histograms of a representative donor showing Ki67 staining in M-CSF and GM-CSF derived macrophages either with HS or FBS. Percentage of positive cells is shown in each case. **(B)** Dot plots representing cell cycle profile showing DNA (7-AAD) and RNA (Pyronin Y) content of M-CSF and GM-CSF differentiated macrophages cultured with HS or FBS and quantified by flow cytometry. Data from a representative donor is shown. **(C)** Gene expression of cell cycle-related genes and SAMHD1 restriction pathway. mRNA levels of *SAMHD1*, *CDK2*, *CDK4* and *CDK6*, all D-type cyclins (*D1*, *D2 and D3*) and the CDK inhibitors p21 (*CDKN1A*) and p27 (*CDKN1B*) was quantified in M-CSF, human serum (white bars) and M-CSF, FBS (black bars), GM-CSF, human serum (light grey bars) and GM-CSF, FBS (dark grey bars) differentiated macrophages. Data is normalized to M-CSF, FBS relative expression. Mean ± SD of 3 independent donors is shown. ** p<0.005; *** p<0.0005. **(D)** Western blot showing protein expression of different cell cycle proteins, SAMHD1 expression and activation and Hsp90 as loading control. A representative donor is shown. M; M-CSF MDM, GM; GM-CSF MDM. **(E)** HIV-1 replication in M-CSF MDM and GM-CSF MDM cultured with human serum (white bars) or fetal bovine serum (FBS) (black bars). Data represent percentage replication relative to M-CSF culture with FBS. Mean ± SD of 3 different donors performed in triplicate is shown. ** p<0.005; *** p<0.0005
(TIFF)

S3 Fig Mouse peritoneal macrophages resemble GM-CSF expression profile. (A) Comparative gene expression of cell cycle-related genes and SAMHD1 in mouse peritoneal macrophages and human M-CSF and GM-CSF macrophages. mRNA levels of CCND2, CCND3, CDK2, CDK4, SAMHD1 and the CDK inhibitors p21 (CDKN1A) and p27 (CDKN1B) were quantified by real time PCR. Relative expression of each gene vs. GAPDH is plotted. Horizontal bars represent mean values. * p<0.05; ** p<0.005; *** p<0.0005; ns, not significant. (B) Western blot showing protein expression in peritoneal macrophages from 3 different mice. Mice protein expression was compared to different protein concentrations of GM-CSF and M-CSF human macrophages in order to evaluate the relative levels of expression for each sample.
(TIFF)

S4 Fig Cyclin D2 restricts HIV-1 replication and proviral DNA formation in GM-CSF macrophages. **(A)** *SAMHD1* expression levels after knockdown of CCND2 and CCND3 expression by siRNA. Relative mRNA expression of *SAMHD1* in M-CSF (white bars) and GM-CSF (black bars) macrophages. *SAMHD1* mRNA was measured by quantitative PCR and normalized to GAPDH expression. Data represents mean ± SD of 3 different donors and is normalized to Mock-transfected M-CSF macrophages. **(B)** Effective knockdown of *CCND2* expression with two different siRNA sequences (siCCND2#1 and siCCND2#2). *CCND2* mRNA was measured by quantitative PCR and normalized to GAPDH expression. Data represents mean ± SD of 3 different donors and is normalized to Mock-transfected macrophages. **(C)** HIV-1 replication in siCCND2 GM-CSF macrophages. Transfected MDM were infected with a VSV-pseudotyped, GFP-expressing HIV-1 and infection measured 72h later by flow cytometry. Data represent percentage replication relative to mock-transfected macrophages. Mean ± SD of 3 different donors performed in duplicate is shown. **(D)** Proviral DNA formation after 16h infection with HIV-1 BaL of GM-CSF macrophages transfected with the indicated siCCND2 sequences or treated with AZT (3 μM) or raltegravir (RAL; 2 μM). Proviral DNA was normalized to mock-treated macrophages. Mean ± SD of 3 different donors is shown. * p<0.05; ** p<0.005; *** p<0.0005.
(TIFF)

S5 Fig Knockdown of cyclin D2 does not affect macrophage function. **(A)** Cytokine expression in GM-CSF siRNA-treated macrophages. Cytokine mRNA expression was measured using a TaqMan Human Cytokine Network array and expression of each gene was normalized to GAPDH. Data is normalized to Mock-transfected macrophages. Mean ± SD of 2 different donors performed in duplicate is shown. **(B)** Induction of cytokine gene expression following LPS (100 ng/ml) treatment in siCCND2 GM-CSF macrophages. mRNA expression of *CCL-2* and *IL-10* was measured by quantitative PCR and normalized to GAPDH expression. Data is normalized to untreated Mock-transfected GM-CSF macrophages. A representative donor is shown.
(TIFF)
