# Supplementary figures and images for: The G1/S Specific Cyclin D2 Is a Regulator of HIV-1 Restriction in Non-proliferating Cells
Source: PLoS Pathog. 2016 Aug 19;12(8):e1005829. doi: 10.1371/journal.ppat.1005829 (PMC4991798; doi:10.1371/journal.ppat.1005829)

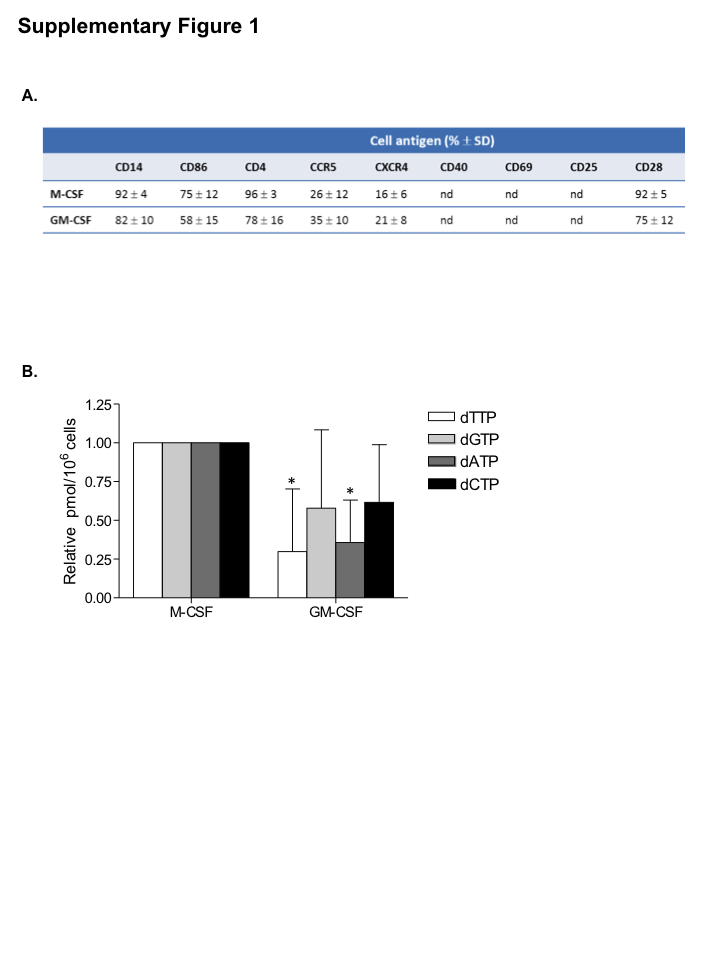

Supplement: S1 Fig — (A) Summary of cell surface antigens expression. Markers of macrophage differentiation, HIV-1 receptor and coreceptors and cell activation and proliferation markers were evaluated by flow cytometry. The table shows mean ± SD of at least 3 different donors. nd, not detected. (B) Intracellular dNTP levels in M-CSF or GM-CSF differentiated MDM. Intracellular dNTPs were extracted from M-CSF (left) or GM-CSF (right) differentiated MDM and dNTP content was determined using a polymerase-based method. dNTP content is highly variable between donors and therefore data is relativized to M-CSF differentiated MDM. Mean ± SD of 3 different donors is shown. * p<0.05 (TIFF) [file ppat.1005829.s001.tiff]

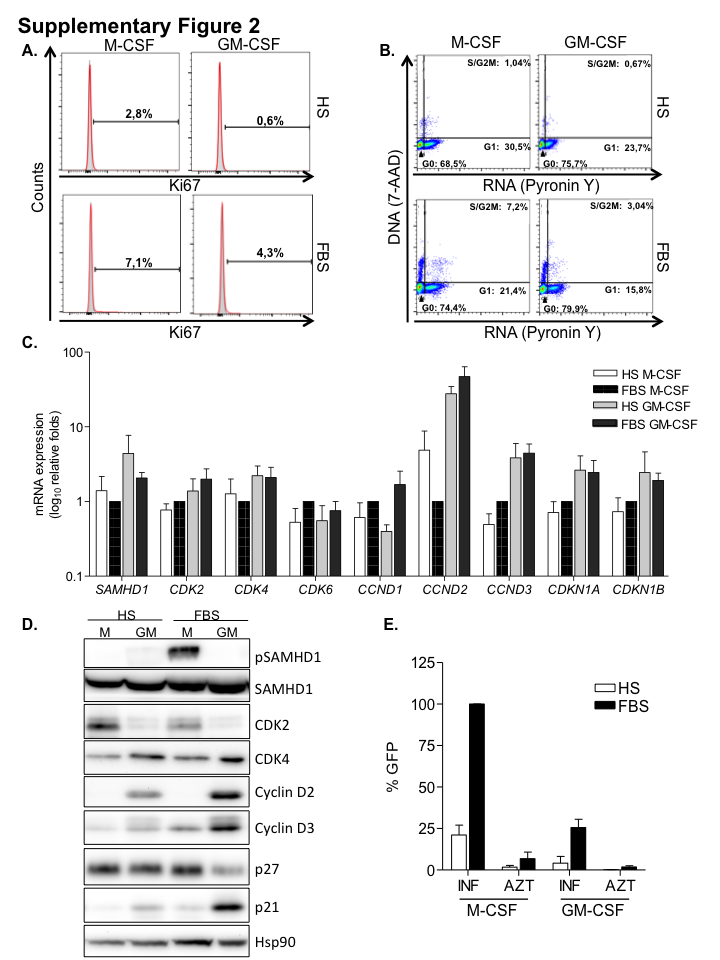

Supplement: S2 Fig — (A) Evaluation of cell proliferation by Ki67 staining. Histograms of a representative donor showing Ki67 staining in M-CSF and GM-CSF derived macrophages either with HS or FBS. Percentage of positive cells is shown in each case. (B) Dot plots representing cell cycle profile showing DNA (7-AAD) and RNA (Pyronin Y) content of M-CSF and GM-CSF differentiated macrophages cultured with HS or FBS and quantified by flow cytometry. Data from a representative donor is shown. (C) Gene expression of cell cycle-related genes and SAMHD1 restriction pathway. mRNA levels of SAMHD1, CDK2, CDK4 and CDK6, all D-type cyclins (D1, D2 and D3) and the CDK inhibitors p21 (CDKN1A) and p27 (CDKN1B) was quantified in M-CSF, human serum (white bars) and M-CSF, FBS (black bars), GM-CSF, human serum (light grey bars) and GM-CSF, FBS (dark grey bars) differentiated macrophages. Data is normalized to M-CSF, FBS relative expression. Mean ± SD of 3 independent donors is shown. ** p<0.005; *** p<0.0005. (D) Western blot showing protein expression of different cell cycle proteins, SAMHD1 expression and activation and Hsp90 as loading control. A representative donor is shown. M; M-CSF MDM, GM; GM-CSF MDM. (E) HIV-1 replication in M-CSF MDM and GM-CSF MDM cultured with human serum (white bars) or fetal bovine serum (FBS) (black bars). Data represent percentage replication relative to M-CSF culture with FBS. Mean ± SD of 3 different donors performed in triplicate is shown. ** p<0.005; *** p<0.0005 (TIFF) [file ppat.1005829.s002.tiff]

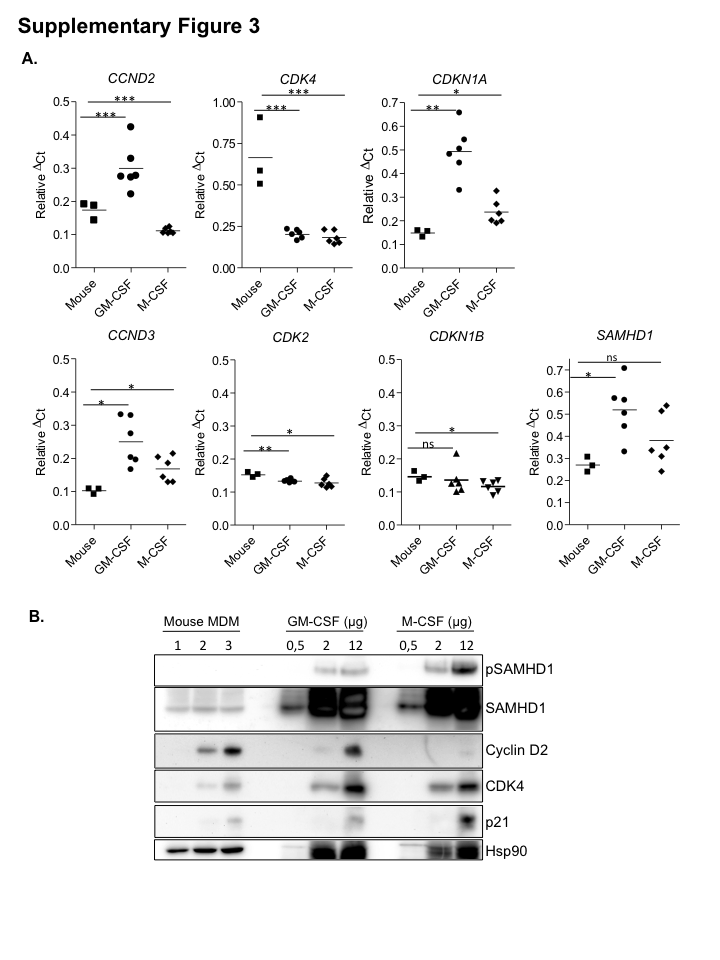

Supplement: S3 Fig — (A) Comparative gene expression of cell cycle-related genes and SAMHD1 in mouse peritoneal macrophages and human M-CSF and GM-CSF macrophages. mRNA levels of CCND2, CCND3, CDK2, CDK4, SAMHD1 and the CDK inhibitors p21 (CDKN1A) and p27 (CDKN1B) were quantified by real time PCR. Relative expression of each gene vs. GAPDH is plotted. Horizontal bars represent mean values. * p<0.05; ** p<0.005; *** p<0.0005; ns, not significant. (B) Western blot showing protein expression in peritoneal macrophages from 3 different mice. Mice protein expression was compared to different protein concentrations of GM-CSF and M-CSF human macrophages in order to evaluate the relative levels of expression for each sample. (TIFF) [file ppat.1005829.s003.tiff]

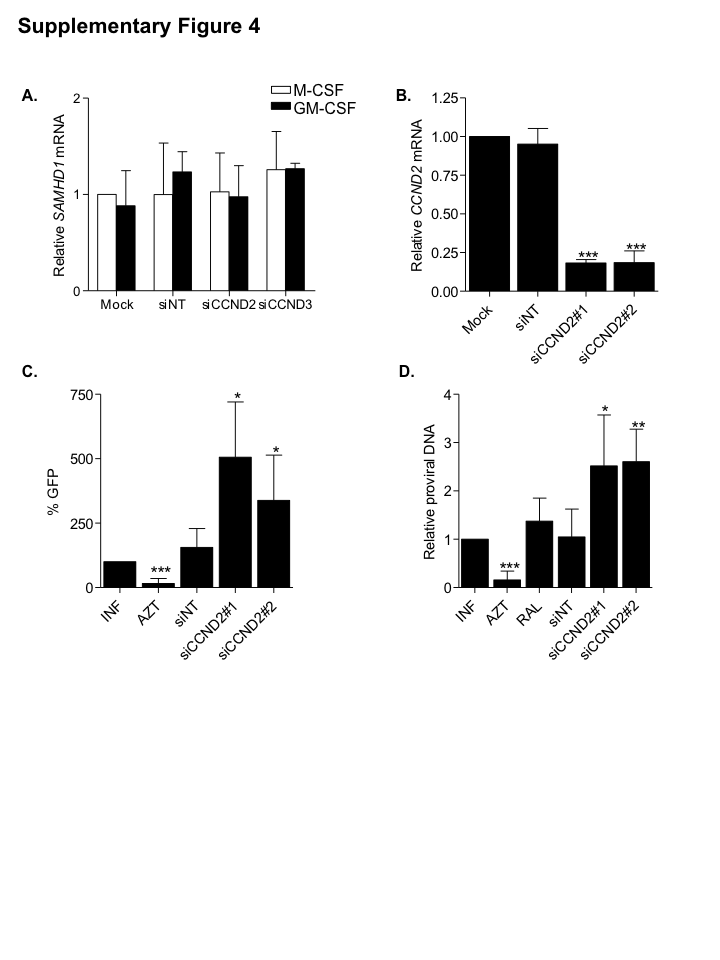

Supplement: S4 Fig — (A) SAMHD1 expression levels after knockdown of CCND2 and CCND3 expression by siRNA. Relative mRNA expression of SAMHD1 in M-CSF (white bars) and GM-CSF (black bars) macrophages. SAMHD1 mRNA was measured by quantitative PCR and normalized to GAPDH expression. Data represents mean ± SD of 3 different donors and is normalized to Mock-transfected M-CSF macrophages. (B) Effective knockdown of CCND2 expression with two different siRNA sequences (siCCND2#1 and siCCND2#2). CCND2 mRNA was measured by quantitative PCR and normalized to GAPDH expression. Data represents mean ± SD of 3 different donors and is normalized to Mock-transfected macrophages. (C) HIV-1 replication in siCCND2 GM-CSF macrophages. Transfected MDM were infected with a VSV-pseudotyped, GFP-expressing HIV-1 and infection measured 72h later by flow cytometry. Data represent percentage replication relative to mock-transfected macrophages. Mean ± SD of 3 different donors performed in duplicate is shown. (D) Proviral DNA formation after 16h infection with HIV-1 BaL of GM-CSF macrophages transfected with the indicated siCCND2 sequences or treated with AZT (3 μM) or raltegravir (RAL; 2 μM). Proviral DNA was normalized to mock-treated macrophages. Mean ± SD of 3 different donors is shown. * p<0.05; ** p<0.005; *** p<0.0005. (TIFF) [file ppat.1005829.s004.tiff]

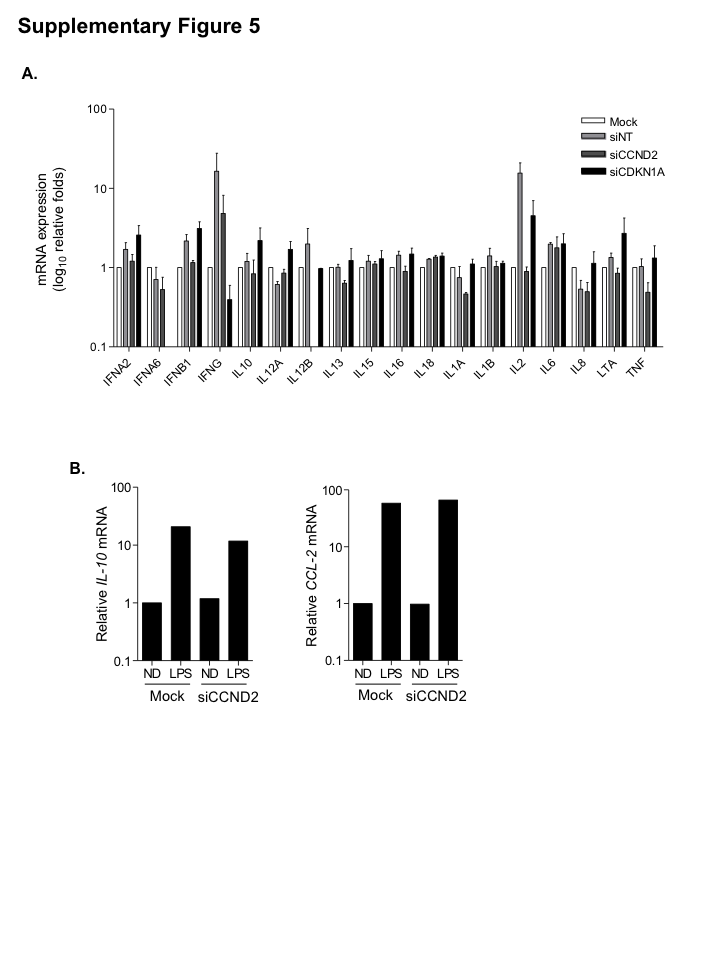

Supplement: S5 Fig — (A) Cytokine expression in GM-CSF siRNA-treated macrophages. Cytokine mRNA expression was measured using a TaqMan Human Cytokine Network array and expression of each gene was normalized to GAPDH. Data is normalized to Mock-transfected macrophages. Mean ± SD of 2 different donors performed in duplicate is shown. (B) Induction of cytokine gene expression following LPS (100 ng/ml) treatment in siCCND2 GM-CSF macrophages. mRNA expression of CCL-2 and IL-10 was measured by quantitative PCR and normalized to GAPDH expression. Data is normalized to untreated Mock-transfected GM-CSF macrophages. A representative donor is shown. (TIFF) [file ppat.1005829.s005.tiff]
